# A Systematic Review on the Efficacy of Vaporized Hydrogen Peroxide as a Non-Contact Decontamination System for Pathogens Associated with the Dental Environment

**DOI:** 10.3390/ijerph18094748

**Published:** 2021-04-29

**Authors:** Rukshana Ahmed, Riaan Mulder

**Affiliations:** Prosthetics Department, Dental Faculty, The University of the Western Cape, Parow, Cape Town 7505, South Africa; rmulder@uwc.ac.za

**Keywords:** hydrogen peroxide, vaporized hydrogen peroxide, bio-decontamination, enumeration, stainless steel

## Abstract

Aerosol generation and a wide range of pathogens originating from the oral cavity of the patient contaminate various surfaces of the dental clinic. The aim was to determine the efficacy of vaporized hydrogen peroxide fogging on pathogens related to the dental environment and its possible application in dentistry. PICOS statement (Population, Intervention, Comparison/Control, Outcome and Study design statement) was used in the review. Six electronic databases were searched for articles published from 2010 to 2020. Articles written in English reporting vaporized hydrogen peroxide on pathogens deemed to be relevant to the dental environment were assessed. The quality of the studies was assessed using the risk-of-bias assessment tool designed for the investigation of vaporized hydrogen peroxide application in dentistry. A total of 17 studies were included in the qualitative synthesis. The most commonly reported single bacterial pathogen was Methicillin-resistant Staphylococcus aureus in five studies, and the viruses Feline calicivirus, Human norovirus, and Murine norovirus were featured in three studies. The results of the studies reporting the log kill were sufficient for all authors to conclude that vaporized hydrogen peroxide generation was effective for the assessed pathogens. The studies that assessed aerosolized hydrogen peroxide found a greater log kill with the use of vaporized hydrogen peroxide generators. The overarching conclusion was that hydrogen peroxide delivered as vaporized hydrogen peroxide was an effective method to achieve large levels of log kill on the assessed pathogens. The hydrogen peroxide vapor generators can play a role in dental bio-decontamination. The parameters must be standardized and the efficacy assessed to perform bio-decontamination for the whole clinic. For vaporized hydrogen peroxide generators to be included in the dental bio-decontamination regimen, certain criteria should be met. These include the standardization and efficacy assessment of the vaporized hydrogen peroxide generators in dental clinics.

## 1. Introduction

Infection control has always been a core objective in dentistry; however, it has risen to greater importance given the SARS-CoV-2 pandemic. Aerosolized viruses and bacteria, such as Tuberculosis, Candida auris, and Staphylococcus aureus, once inhaled by either patient or healthcare worker, can result in far-reaching health consequences [1,2,3].

Infected droplets can be spread by dental instruments from the mouth of the dental patient, such as high-speed rotating handpieces and ultrasonic devices. The contaminated aerosol settle on exposed surfaces, resulting in environmental contamination. Virus or bacterial pathogens may survive on inanimate surfaces for prolonged periods of time and, as a result, serve as reservoirs for cross-contamination. These aforementioned surfaces increase the risk of transferring pathogens to patients through hand contact [3]. The surfaces most frequently touched in the dental environment include switches/buttons on the dental chair, dental light, radiography equipment, dental material containers, dental curing lights, lasers, and computer equipment. Despite surface disinfection protocols, many of the inanimate objects are not routinely disinfected and together with the hands of staff become vectors for transmission of healthcare-associated infections [3,4]. Studies determined that established disinfection methods showed their inability to eliminate environmental contamination of certain pathogens that are associated with direct transmission [5]. In addition, an increase in the bacterial load after the use of a neutral detergent was reported. A neutral detergent is composed of surfactants, fillers, and chelating agents. They are typically not used in pathogen eradication [6]. Enhanced cleaning that deviates from traditional surface cleaning (traditional surface cleaning or terminal cleaning aims to reduce the number of pathogens on surfaces to reduce transmission) not only reduced the bacterial load in the environment but also reduced the number of organisms on the hands of staff. Disinfection procedures that involve physical contact with the surfaces (spray, wipe, and spray techniques) are widely used but are usually labor-intensive and not always effective, as it is impossible to reach all hidden surfaces. For this reason, it is imperative to investigate the efficacy and then adopt other infection control approaches to decontaminate the dental environment between patients and minimize the risk of transmission of diseases [5,7].

As a result of this need for increased efficacy of decontamination for dentistry, there is potential in the use of gaseous decontamination either on its own or after conventional cleaning. Vaporized hydrogen peroxide (VHP) can be a suitable decontaminant as it is effective for the in vitro inactivation of enveloped and non-enveloped viruses [4,8]. Hydrogen peroxide is an effective biocide in its gaseous (vaporized and aerosolized) form against viruses, spores, fungi, and bacteria [1,5]. The vaporized solution of hydrogen peroxide, which is based on water, is activated by plasma and acts as an oxidizing and disinfecting agent when it settles and contacts the surfaces of all objects in the room. In doing so, it attacks essential cell components, such as the DNA, lipids, and protein of the pathogen cell wall [1].

There are various terms used in the literature used to describe hydrogen peroxide vapor, vaporized hydrogen peroxide (VHP), and aerosolized hydrogen peroxide (aHP). Hydrogen peroxide vapor has been used effectively to decontaminate enclosed areas, like incubators, medicine trolleys, laboratory cabinets, operating rooms, isolation room, general medical wards, and intensive care units. The air particles in the room assist with spreading the hydrogen peroxide vapor [1,7,9,10]. This process is relatively non-toxic for humans, the environment, and medical materials/devices because hydrogen peroxide vapor degrades into water and oxygen with no residue typically found [5,7,8]. The importance of the compatibility between materials used in the dental environment with physical or liquid chemical germicides should not be overlooked.

VHP generators are no-touch decontamination and therefore circumvent problems associated with operators during manual disinfection such as incorrect application and use of cleaning agents. Due to its vapor form, it can disinfect all objects that are in contact with the air as well as hard to reach places on inanimate objects [5,8]. Whole-room disinfection using conventional surface cleaning followed by VHP was found to be highly effective in reducing the level of aerobic bacterial contamination below detectable counts [1,6]. An additional advantage is that, while the hydrogen peroxide is being dispensed by a portable vapor generator into a vaporized state for decontamination, staff do not need to remain in the room, thus, providing an opportunity to allocate staff to other tasks or continue treatment in another room. The greatest disadvantage of this form of decontamination is that the generator set-up and the preparation process have an initial learning curve and must be operated by trained personnel [1].

Depending on the generator utilized it could be time-consuming and expensive to ensure the appropriate hydrogen peroxide concentration and validation are achieved [7,10]. Additionally, the room must be vacated and possibly pre-cleaned to remove visible contamination. VHP can irritate eyes, mucous membranes, skin, and lungs—if it is inhaled. For this reason, the room requires a venting stage to achieve a safe level of VHP before staff may enter the room [1].

The efficacy of surface decontamination and techniques in dental environments has been questioned, especially in our current climate of SARS-CoV-2 hypervigilance. Contaminated hands can result in self-inoculation and act as a route for disseminating pathogens. No-touch decontamination methods can, therefore, play a pivotal role in the prevention of viral transmission in the dental environment.

## 2. Objective

In this review we aim to determine the efficacy of VHP fogging on pathogens related to the dental environment and its possible applications in dentistry.

## 3. Methods

We reported this review following the Preferred Reporting Items for Systematic Reviews and meta-analyses (PRISMA) statement [11]. A meta-analysis and associated plots/graphs were not completed for this systematic review.

Research questions:

Efficacy of VHP fogging against dental environment pathogens:

− To what log kill are the pathogens that play a role in dentistry eliminated by VHP fogging?
− What VHP fogging disinfection methods could be effective in the dental environment?

### 3.1. PICOS Statement

People/participants = Pathogens on surfaces exposed with VHP

Intervention/Event = VHP fogging

Comparison = Surface disinfection

Outcome = Bio-decontamination in terms of log kill

Study design = Quasi-experimental study design

### 3.2. Search Strategy

The following electronic databases were searched to identify articles reporting results on VHP fogging for pathogens that can contaminate the dental environment using terms with Boolean operators published between January 2010 And October 2020 (Table 1): DOAJ, Ebscohost, Pubmed/Medline, Scopus, Sceilio, and Web of Science. Studies were limited to articles written in the English language.

### 3.3. Eligibility Criteria

Inclusion criteria: Studies (2010–2020) published in English that investigated the efficacy of the VHP fogging on pathogens that can contaminate the dental environment were considered for inclusion. The following original research articles: Articles published in peer-reviewed, scientific journals and research conducted in dental-care or in vitro settings that implemented a no-touch disinfection method with hydrogen peroxide against any pathogen that presented with the possibility of being a contaminating pathogen from patients to the dental environment and staff were considered for inclusion.

Exclusion criteria: Articles, such as editorials, commentaries, non-peer-reviewed articles, systematic reviews, scoping reviews, pathogen outbreaks, conference papers, and surveillance reports, were excluded. Studies in which the pathogen assessed against hydrogen peroxide was not able to contaminate the dental environment were ignored including studies that evaluated VHP as an adjunct to service cleaning.

### 3.4. Study Selection

Two reviewers independently assessed the eligibility of the searched studies. The titles and abstracts were primarily screened to identify whether the criteria were met. The full texts of selected studies during primary screening were reviewed for the final study selection. Any discrepancies were resolved by sharing opinions and consultation with the other author, if necessary.

### 3.5. Data Extraction

After data extraction, two reviewers independently extracted data, such as information on the pathogen used and on what type of surface, outcome level (log kill), the methodology of use including manufacturer instructions. Included also was the control measures in terms of study standardization, the parameters of the hydrogen peroxide in parts per million, monitoring of the concentration, and whether the study was transferable to the dental environment due to the results and pathogen used.

### 3.6. Control Measures

The general control measures considered were the use of a biological indicator (BI) or electronic forms to assess the efficacy and consistency of the VHP fogging, the concentration of the VHP reached in the area, and the dwell time. Furthermore, environmental control of the fogged area to receive the vapor and the efficacy of not contaminating the assessed samples at any stage of the research to achieve accurate data collection.

### 3.7. Quality Assessment

The quality of studies was assessed using a critical appraisal tool that was developed by two reviewers (R.A. and R.M.). The decision to formulate an independent tool for this study was based on the specific aspects of hydrogen peroxide that were being investigated. The key domains that were evaluated were decontamination by hydrogen peroxide and whether this can be translated into clinical practice, including dentistry. Studies that were unclear with their objectives and did not meet the aims of the study, and were excluded.

If the studies did not use standard bacterial strains and control pathogens they were eliminated. Studies that used surface cleaning as an adjunct to VHP decontamination were excluded as well. If articles were not clear on where they fall within the context of the criteria, they were discussed between the two reviewers, and, if necessary, an agreement was reached with the corresponding author. Differing opinions were, therefore, discussed between the two reviewers and a consensus was reached.

The specific information assessed under each domain was as follows:

(1) selection of participants included the pathogen exposed on the surface with VHP; (2) confounding variables included the correct percentage of VHP used for the machine and the equipment used per the manufacturer instructions with appropriate monitoring of the VHP parts per million and dwell time; (3) VHP exposure measurement included how the study was standardized to ensure the validity of all the specimens being treated the same and preventing contamination; (4) blinding for outcome assessment included if the authors were blinded to what sample is the control or the test specimen. This determined if the study was a quasi-experimental study design; (5) incomplete outcomes included if there were any failed samples or where the experiment could not be completed or outlier results detected, and (6) selective outcome reporting where not all the data were presented or data presented not in terms of the log kill. The results for the assessment of quality were displayed using Review Manager (RevMan) version 5.3 software (The Cochrane Collaboration, Oxford, UK).

## 4. Results

### 4.1. Search Results

**Figure 1:**
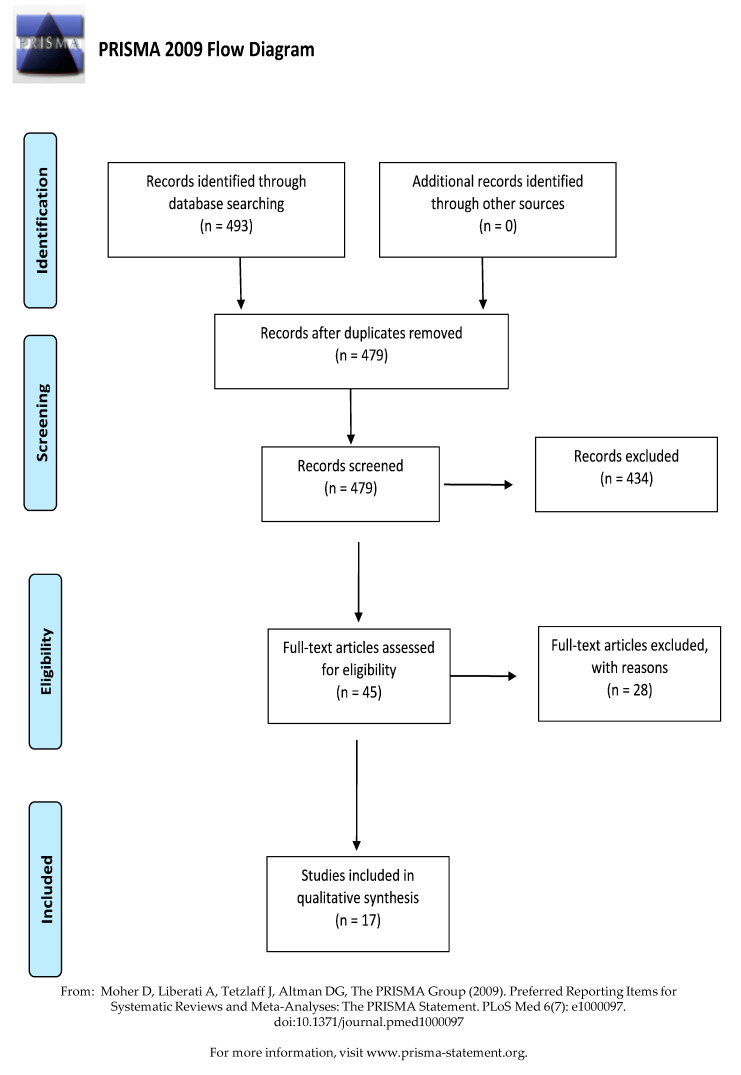
PRISMA flow diagram of the study selection.

A total of 493 studies were retrieved from six databases. The duplicate records were removed (*n* = 479), and the eligibility criteria were applied for the selection process. After reviewing the full text, 434 articles were excluded for the following reasons: irrelevant for the research topic (*n* = 345), unavailable full-text (*n* = 3), review articles (*n* = 24), pathogens were not transferable to dentistry (*n* = 11), and VHP as an adjunct to surface cleaning (*n* = 40). Finally, 17 articles were included in this review (Figure 1).

### 4.2. Characteristics of the Included Studies

The characteristics of the eligible studies are presented in (Table 2). The publication distribution was between 2010 and 2020. The majority of the articles were completed in the United Kingdom (*n* = 8), Sweden (*n* = 3), and the USA (*n* = 2), followed by Brazil (*n* = 1), France (*n* = 1), Germany (*n* = 1), and The Netherlands (*n* = 1).

### 4.3. Risk of Bias in the Included Studies

**Figure 2:**
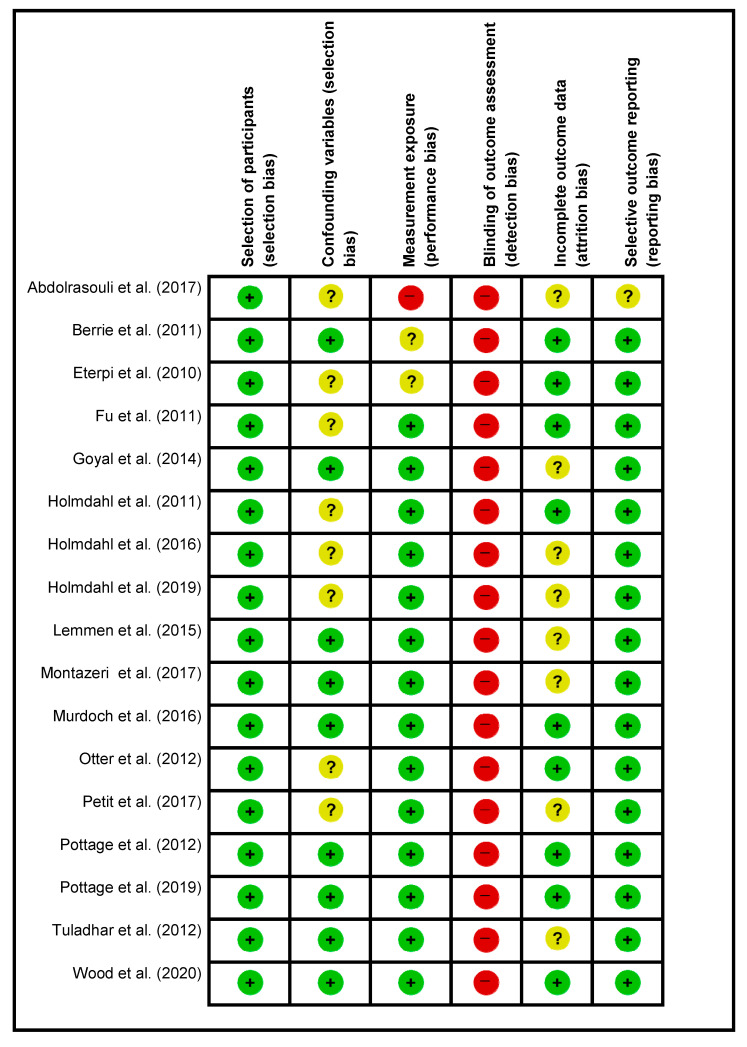
Risk of bias summary.

The quality of the 17 selected studies was assessed, and the results are summarized in Figure 2. All the studies were of high risk for detection bias concerning blinding toward the outcome of pathogen assessment as the samples were not anonymized when sent for pathogen assessment. One study presented with an unclear risk for incomplete data representation or outlier results being present [12]. Confounding variables for selection bias was of unknown risk for eight of the articles [12,13,14,15,16,17,18,19]. Incomplete data presentation leading to attrition bias or at least the risk of misinterpretation of the log loss of viruses were not determined due to the various steps of virus remuneration after VHP exposure [12,14,15,17,18,19,20,21,22,23,24].

### 4.4. Characteristics of VHP Decontamination on Pathogens

The characteristics of VHP decontamination are presented in Table 2 and Table 3. Bioquell was the manufacturer of 13 of the 19 machines assessed in the selected studies [12,14,15,16,17,18,19,21,22,23,25,26,27]; however, irrespective of the VHP unit used, all the studies reported favorable outcomes (towards the VHP generators rather than the aerosolized hydrogen peroxide (aHP) generators) for log reduction of the assessed pathogens. Methicillin-resistant Staphylococcus aureus (MRSA) was the most used bacterial pathogen in five studies and with the viruses Feline calicivirus, Human norovirus, and Murine norovirus featured in three studies.

Most of the VHP generators are equipped with monitoring systems, but part per million (ppm) monitoring is essential to ensure the desired concentration of hydrogen peroxide is reached for the desired dwell time. Additionally, the use of standardized validated Geobacillus stearothermophilus biological indicators [14,15,16,17,21,22,23,25,28], are important to set the benchmark for the efficacy of the VHP with the manufacturer’s instructions. The surface that received the pathogen was predominantly stainless steel in the form of discs, coupons, or tape with the exceptions of cell culture well plates [12,16,17] and cryogenic tube caps [19]. Two authors used stainless steel as well as some additional materials [20,27]. The log kill was sufficient for all the authors to conclude that VHP generation was effective for the assessed pathogens. The studies that assessed aHP found a greater log kill with VHP generators [14,15].

## 5. Discussion

Stainless steel in its nature is resilient and is a common material found in the clinical areas of medicine and dentistry. In this review, stainless steel discs were the most commonly used and preferred carrier or medium for inoculation with a bacterium, virus, or fungi. A 10 mm diameter disc was used by 5 of the 17 included studies and four studies used unspecified stainless-steel disc. This evidence from the aforementioned five studies is therefore translatable to dentistry due to the high contact surfaces in a dental clinic containing some kind of stainless-steel surface.

Some studies did not report on the hydrogen peroxide liquid and/or the percentage used in the machines [12,15,16,17,18] nor on the model of the machine [12]. The hydrogen peroxide liquid and percentage cannot be assumed, as some liquids are optimized with additives for certain machines. The concentration of the hydrogen peroxide liquid plays a role in the time period that the machine needs to be on in order to generate the appropriate parts per million in the room. Some authors additionally did not indicate in their articles if the manufacturer’s instructions were followed. The combination of the unknown run time of the machine and unknown liquid concentration makes certain studies difficult to reproduce. Although the exposure time, dwell time, and the grams of hydrogen peroxide used per cubic meter are essential information, the ability to replicate a study requires more information [12,15,16,17,18].

Attrition bias is an important component of microbiological studies and although the studies utilized positive controls that were not exposed to hydrogen peroxide vapor, the loss of pathogens during the methodology influences the results and leads to increased attrition bias. The bias within the study as a result of surviving enumerated pathogens after VHP is purely concerning the control pathogens not exposed to VHP but exposed to similar periods before enumeration. On the other hand, the determination of the possible enumerated pathogens at every step ensures that the authors can explain certain results. This was evident, e.g., as H_2_O_2_ vapor unexposed *C. auris* was able to survive in a desiccated state in vitro after 4 weeks, whereas non-*C. auris* had reduced viability (data was not shown by the authors) [12].

Certain studies recognized the loss of the pathogens during the methodology and reported on the log reduction determined in the studies for viruses [16,24,25] and bacteria [13,26]. The loss of pathogens has been specifically noted with viruses [25] but has also occurred with bacteria [13] on dry surfaces indicated at each step of the methodology with reconstitution and neutralization. The assessment of the lost pathogens provides transparency to the research. For review purposes, a greater opportunity for result comparison with other studies and duplication of the methodology can be achieved. Other positive, negative and additional control measures were employed by authors [27,28]. These measures provided greater validity to the methodology and less possible bias.

The appropriate sample size to receive hydrogen peroxide vapor (H_2_O_2_ vapor) in conjunction with the biological indicator next to the sample assessed confirms the H_2_O_2_ vapor efficacy to eliminate the pathogens on a biological indicator. The importance of such a measure is evident when the selective outcome reporting (reporting bias) is noted. In a study by [12], one Indian (the United Kingdom outbreak unrelated) *C. auris* isolate grew repeatedly in two out of the triplicate wells exposed to H_2_O_2_ vapor. These authors concluded that some isolates may be more resilient to this form of disinfection [12]. Geobacillus stearothermophilus is the verified bacterial pathogen used on the stainless-steel discs as a biological indicator for the efficacy of bio-decontamination of hydrogen peroxide vapor generators by the machine manufacturers. The biological indicator pathogen elimination from the stainless-steel discs after hydrogen peroxide vapor is easier to achieve with high numbers of log kill, compared to hydrogen peroxide vapor systems on Methicillin-resistant Staphylococcus aureus (MRSA) [22,28]. The elimination of MRSA by 35% hydrogen peroxide vapor is the requirement for VHP, rather than 5% and 10% concentrations [23]. MRSA is quite resilient compared to the biological indicators and is a suitable parameter to assess the efficacy of VHP for dental purposes. Additionally, the studies that presented the growth medium containing additional serums added to the simulation of surface contamination.

These additional growth mediums present a greater challenge to the VHP bio-decontamination process [14,16,17,18,21,26,27]. This applies to dentistry, as the pathogens will be present in saliva, blood, and water as organic and inorganic matter. Therefore, surface contamination from the serum poses a real-life challenge to the hydrogen peroxide vapor articles assessed in this systematic review. The result was that VHP generators with a 35% H_2_O_2_ are the most appropriate machine for dentistry; however, the parameters should be defined in more detail than is presented in the 17 reviewed articles to align the methodologies toward consistent and predictable bio-decontamination.

## 6. Conclusions

The overarching conclusion is that H_2_O_2_ delivered as VHP was an effective method to achieve large levels of log kill on the assessed pathogens. Within the limitations of all the studies’ parameters, including the presentation of no blinding as well as pathogen log loss during the methodology, VHP was found to be a suitable form of bio-decontamination. Head-to-head direct comparison of results between articles was not possible due to the heterogeneity in the methodologies. The essential methodology benchmarks should include the presentation of the dwell time, parts per million, and the initial concentration of the H_2_O_2_. Due to the heterogeneity of the methodologies, evaluating each article concerning the bias selection criteria was the only effective way to determine the relevance to dentistry.

All the articles have applications to dentistry bio-decontamination. They showed the efficacy of VHP in spaces and surfaces similar to a dental clinic. Further investigation of VHP in dental clinics is required with certain variables that must be known and standardized to assure the validity and reproducibility regarding the H_2_O_2_ concentration, dwell time, and a constant ppm or defined ppm range during the dwell time. The enumerated pathogens at every step of the methodology, from inoculation on the test surface to the enumeration of the exposed and unexposed samples, should be completed. This safeguard will ensure the correct determination of the log loss of pathogens. From the results of the reviewed articles, a statistically calculated sample size performed in triplicate should be standardized.

## Figures and Tables

**Table 1:** Search terms for database searches.

Database	Search Terms
DOAJ	Hydrogen peroxide vapor OR Hydrogen peroxide vapor OR vaporized hydrogen peroxide (VHP), producing system OR Hydrogen peroxide fogger OR hydrogen peroxide in vitro AND ( decontamination or disinfection )
Ebscohost	Hydrogen peroxide vapor OR Hydrogen peroxide vapour OR VHP producing system OR Hydrogen peroxide fogger OR hydrogen peroxide in vitro AND (decontamination or disinfection )
Pubmed	(((((Hydrogen peroxide vapor) OR (Hydrogen peroxide vapour)) OR (HPV producing system)) OR (Hydrogen peroxide fogger)) OR (Hydrogen peroxide fog)) AND (decontamination)
Scopus	(((((Hydrogen peroxide vapor) OR (Hydrogen peroxide vapour)) OR (HPV producing system)) OR (Hydrogen peroxide fogger)) OR (Hydrogen peroxide fog)) AND (decontamination)
Sceilio	(((((Hydrogen peroxide vapor) OR (Hydrogen peroxide vapour)) OR (HPV producing system)) OR (Hydrogen peroxide fogger)) OR (Hydrogen peroxide fog)) AND (decontamination)
Web of Science	(Hydrogen peroxide vapo?r* OR HPV producing system* AND Enterococcus faecalis AND Candida albicans AND decontamination)

**Table 2:** The characteristics of the included studies for VHP decontamination (*n* = 17).

Characteristics	*n* or *n* (%)
Publication Year	*n* = 17
2010	1 (0.05)
2011	3 (0.17)
2012	3 (0.17)
2014	1 (0.05)
2015	1 (0.05)
2016	2 (0.11)
2017	3 (0.17)
2019	2 (0.11)
2020	1 (0.05)
Location	*n* = 17
United Kingdom	8 (0.47)
Sweden	3 (0.17)
USA	2 (0.11)
Brazil	1 (0.05)
France	1 (0.05)
Germany	1 (0.05)
The Netherlands	1 (0.05)
Hydrogen Peroxide Vapourizing machine	(*n* = 19) or *n*% of total machines
Aeroclave	1 (0.05)
Bioquell	1 (0.05)
Bioquell BQ-50	1 (0.05)
Bioquell Clarus C	2 (0.10)
Bioquell Clarus L	1 (0.05)
Bioquell Clarus R	3 (0.15)
Bioquell Clarus S	1 (0.05)
Bioquell Q10	4 (0.21)
Liquid Verne Veiling equipment	1 (0.05)
Sterinis aHP	1 (0.05)
Steris La Calhene VHP	1 (0.05)
Steris VHP	1 (0.05)
Sterinis system SR2	1 (0.05)
Assessed Pathogen:	*n* or *n*% of total pathogens
Candida	(*n* = 34)
Various *Candida species*	34 (100)
Bacteria	(*n* = 27)
*Acholeplasma laidlawii*	1 (0.03)
*Acinetobacter baumannii*	1 (0.03)
*Bacillus anthracis (Ames) spores*	1 (0.03)
*Brucella abortus*	1 (0.03)
*Burkholderia pseudomallei*	1 (0.03)
*Clostridium difficile*	1 (0.03)
*Escherichia coli*	1 (0.03)
*Geobacillus stearothermophilus* biological indicators	9 (0.33)
MDR *Acinetobacter baumannii*	1 (0.03)
Methicillin-resistant *Staphylococcus aureus* (MRSA)	5 (0.18)
*Mycoplasma pneumoniae*	1 (0.03)
*Mycoplasma gallisepticum*	1 (0.03)
*Mycobacterium tuberculosis*	1 (0.03)
*Vancomycin- resistant Enterococcus* (VRE)	1 (0.03)
*Yersinia pestis*	1 (0.03)
Virus	*n* = 21
*Adenovirus*	2 (0.09)
*Avian influenza virus* (AIV)	1 (0.04)
*Escherichia virus MS2*	1 (0.04)
*Feline Calicivirus*	3 (0.14)
*Foot and mouth disease* (FMDV)	1 (0.04)
*Human adenovirus type 1*	1 (0.04)
*Human norovirus*	3 (0.14)
*Influenza A virus* (H1N1)	1 (0.04)
*Murine norovirus* (MNV)	3 (0.14)
*Pseudomonas virus phi6*	1 (0.04)
*Poliovirus*	1 (0.04)
*Rotavirus*	1 (0.04)
*Swine influenza virus* (SwIV)	1 (0.04)
Transmissible *Gastroenteritis coronavirus* of pigs (TGEV)	1 (0.04)
Characteristics:	
Method of inoculation	*n* = 29, *n*% of total surfaces
Sabouraud’s dextrose agar and fabric	2 (0.06)
Stainless steel 10 mm-diameter discs/coupon	5 (0.17)
Stainless steel 3 mm-diameter discs/coupon	1 (0.03)
Stainless steel 2.2 cm × 2.5 cm disc	1 (0.03)
Tyvek-pouched stainless steel disc/coupon	5 (0.17)
Plastic plates	2 (0.06)
Steel embossing tape 2.5 cm × 5 cm	1 (0.03)
Roller bottle	1 (0.03)
Unspecified stainless steel/coupon	4 (0.14)
Gauze	1 (0.03)
Glass	1 (0.03)
Painted joint tape	1 (0.03)
Wood	2 (0.06)
Ceramic tile	1 (0.03)
N95 Filter medium	1 (0.03)
Efficacy: Log kill	
>8 log	1
>6 log	4
>4 log	4
>3 log	2
<2 log	2
1.3–3.5 log reduction	1
Log reduction not specified	4

**Table 3:** Summary of items regarded in the risk assessment of the chosen article.

Author	Country of Study	Aim/Objective	Pathogen Used	Methodology:-Hydrogen Peroxide Concentration	Blinding and Controls	Sample Hanling and Contamination Prevention	Failed Experiments and Data/Results Not Presented	Pathogens Placed on Material	Outcome (Level of Bio-Decontamination)
A Abdolrasouli et al. 2017	United Kingdom	In vitro evaluation of the efficacy of VHP on standard and outbreak *C. auris.*	34 different yeast isolates: 4 strains (*Candida albicans*, *Candida tropicalis*, *Candida krusei*, *Candida parapsilosis*) 28 outbreak isolates of *C. auris*	Bioquell machine, No H_2_O_2_ liquid concentration, performed following manufacture instructions. 8 g of H_2_O_2_/m^3^.	No blinding. One *C. aurus* control plate with no exposure to VHP. Six yeast-free control wells. No BI used. Done in triplicate. Wells of pathogen grown in a 96-well plate and desiccated, sealed, and kept at 4 °C until exposure to VHP. Viability was then assessed on SDA with *C. auris* control plate.	Did not state how long after fogging well plates were closed to prevent contamination.	One Indian *C. auris* and a specific Indian strain not named. Non-exposed *C. auris. Candida species* and VHP exposed C. aurus survive in a desiccated state. Data not shown.	Well plates.	Data provided evidence that *C. auris* (and other *Candida species*) are effectively killed with a 96.6–100% by H_2_O_2_ vaporization.
E Berrie et al. 2011	United Kingdom	In vitro efficacy of inactivation of recombinant adenovirus by VHP.	Dried recombinant *adenovirus * (Ad5GFP)	Bioquell Clarus S machine, 60 mL of 30% H_2_O_2_ liquid concentration, performed dwell time 45 min. Whole VHP cycle 3 h	Exposed and non-exposed samples to VHP. BI (Biological Indicator) indicators used. The experiment is one disc per dilution and repeated in triplicate	Immediately after the experiment, the samples were transferred to a sterile microbiological safety cabinet.	One to two logs of pathogen lost due to drying or recovery method compared to wet reference samples compared to experiment two at the titer. Viability reduction data explained in the article.	Stainless steel 10-mm-diameter discs.	Data provided evidence that Adenovirus are effectively killed with a 7.6 to 9.4 log kill by H_2_O_2_ vaporization.
M Eterpi et al. 2010	France & United Kingdom	In vitro evaluation of the efficacy of VHP and cold VHP sterilization against *Mycoplasma.*	*Mycoplasma gallisepticum, M. pneumoniae*, and *A. laidlawii*	VHP100 Steris machine. 30% H_2_O_2_ liquid concentration. Three cycles 1200 ppm/15 min; 400–500 ppm/60 min; 180–200 ppm/4 h.	No blinding. VHP unexposed samples kept under a laminar flow hood in sealed Petri dishes for the same time cycle and managed the same as exposed coupons. No BI used. Six treated samples with each method and repeated for times.	Samples were transferred to an SP4-glucose broth immediately after VHP exposure.	Less than one log of pathogen lost due to drying or recovery method as described by Nagatomo et al. 2001 with loss due to recovery ≤0.5 log. Neutralization an additional ≤0.5 log. Viability reduction data explained in the article.	Stainless steel coupons of 1 cm × 3 cm.	Data provided evidence that Mycoplasma is effective with a >4 log kill by H_2_O_2_ vaporization.
T. Y. Fu et al. 2011	United Kingdom	Compare the efficacy, efficiency of VHP and aHP.	Methicillin-resistant Staphylococcus aureus (MRSA), Clostridium difficile and Acinetobacter baumannii.	Bioquell Clarus R machine. 30% H_2_O_2_ liquid. SR2 Sterinis machine with a 5% H_2_O_2_ liquid and silver ion (50 ppm) and orthophosphoric acid (<50 ppm), dose 6 mL/m^3^ recommended by the manufacturer.	No blinding. Both exposed and non-exposed to VHP. BI used. Four cycles per machine with each cycle consisting of three unexposed VHP/aHP and three dry VHP/aHP discs for water, 3% BSA (Bovine Serum Albumin) and 10% BSA. The control was cycled separately over four cycles.	Did not state how long after fogging discs were transferred to prevent contamination, nor the overnight drying to prevent contamination.	No pathogens lost or contaminated samples were described or considered in the methodology. All data presented.	Stainless steel discs with a diameter of 10 mm.	The VHP system achieved a greater level of biological inactivation between 4–6 log for most locations than the aHP system 1–5 log depending on the pathogen.
Goyal et al. 2014	United Kingdom	Evaluate the in vitro efficacy of three volumes of VHP on selected viruses with surface contamination.	FCV as a surrogate of *human norovirus*, TGEV as a surrogate for the SARS virus, *human adenovirus* type 1, AIV (A/chicken/Maryland/ 2007[H9N9]) and SwIV (A/swine/Minnesota/2010 [H3N2]).	Bioquell Clarus L machine. 35% H_2_O_2_ liquid. Hydrogen peroxide at 2 mL/min for 1, 2, 5 min followed by 1.5 mL/min or 15 min equating to the following different volumes: 25, 27, and 33 mL with the treatment time between 2–3 h for the completed cycle.	No blinding. Non-VHP exposed inoculated discs at room temperature. Four BI were exposed to the VHP in corners of the environmental chamber. Positive BI control was not exposed to VHP. 8% Fetal Bovine Serum (FBS) served as soiling present in the culture medium. Each experiment had inoculated discs exposed to each vaporized volume of VHP and one disc not exposed to VHP.	Discs are left to dry in a biosafety cabinet to prevent contamination. After VHP the discs including the non-exposed control discs were transferred immediately to the environmental chamber for titration.	Data was determined concerning the control disc. This allows direct comparison to the test and control discs having the same log loss, making the comparison more accurate. But also leads to not knowing what the log loss of viral load is. Loss of virus log particles during the methodology of drying and recovery. Not calculated	10 mm stainless steel discs.	VHP was virucidal for viruses assessed dried on surfaces, suggesting that VHP can be considered for the disinfection of virus-contaminated surfaces based on the 8% FBS surface contamination.
Holmdahl et al. 2011	Sweden	Comparison of VHP and aHP to BI in various locations.	BI with *G. stearothermophilus*	Steris VHP machine. 5% H_2_O_2_ liquid. 6 mL/m^3^ with 100–150 ppm. Bioquell Q10 machine. 35% H_2_O_2_ liquid. 900 mL per test and results in 6.6 g/m^3^. 338 ppm peak, 3 h.	No blinding. BI was used as control.	BI in Tyvek pouches	Direct comparison of two machines on BI. All results presented.	BI stainless steel disc placed in various locations in the room.	All results presented for the same areas assessed for the two machines. VHP showed a 100% negative result while aHP presented with multiple positive results, the inconsistency with the aHP was 10% kill (100 ppm) followed by two cycles of 79% kill, with the ppm in cycles 2 and 3 being 130 and >150 ppm respectively.
Holmdahl et al. 2016	Sweden	Evaluate the efficacy of VHP in six locations for two virus pathogens with surface contamination.	FCV, feline permissive cell line (FCWF). MNV and permissive murine cell line (RAW 264.7)	Bioquell Q10 machine. As per the manufacturer. No H_2_O_2_ liquid concentration. Gassing time 40–50 min, dwell time 15 min. VHP ppm range 474–505 ppm with a total cycle time of 3 h.	Virus prepared in triplicate in well plates. Two inoculated plates and BI not VHP exposed two areas of the control room. BI exposed at all the positions with VHP next to virus inoculated plates. Each VHP exposure experiment was repeated in triplicate.	Well plates left to dry at room temperature under a hood for 2 h and stored.	Loss of virus log particles during the methodology of drying and recovery were calculated.	Well plates	VHP was virucidal for viruses assessed dried on surfaces, suggesting that VHP can be considered for the disinfection of virus-contaminated surfaces based on the 10% FBS surface contamination.
Holmdahl et al. 2019	Sweden	Assess *norovirus* viability of cytopathic the effect after VHP.	Two *human norovirus* field strains, genogroup I and II. *Murine norovirus*.	Bioquell Q10 machine. No H_2_O_2_ liquid concentration. 860 ppm VHP for 33 min gassing and 55 min dwell. This resulted in 205 g of H_2_O_2_ used.	No blinding. BI and mock samples with no VHP exposure.	Virus samples dried in 35 mm diameter wells of six-well plates mock and VHP treated samples.	Data was determined concerning the lowest detection limit of 10^−0.5^. This allows direct comparison to the test and control discs having the same log loss, making the comparison more accurate. But also leads to not knowing what the log loss of viral load is.	Well pates	BI deactivated and *norovirus* log 5 kill.
Lemmen et al. 2015	Germany	Efficacy of VHP on five pathogens dried onto various hard surfaces.	MDR MRSA and MDR VRE, MDR *A baumannii*. BI as proxy for *D. difficile*	Bioquell Q10 machine. 30% H_2_O_2_ liquid. Three cycles were performed. The dose of 11.2 g/m^3^ achieved after 50–52 min until hydrogen peroxide was 500–600 ppm. 20 min dwell time.	No blinding. BI used. Four of each material inoculated with the pathogen and distributed in four locations exposed to VHP and the same number not exposed to VHP as controls. BI placed in 4 corners of the room and 3 challenge locations.	Kept on a sterile basis until experiment and after VHP exposure transferred to a sterile glass tube with 1 mL distilled water.	Lost pathogens are known and presented in the article and mean log reduction is calculated.	Stainless steel discs, gauze	VHP inactivated all spore BI (>6 log_10_ reduction), and no MRSA, VRE, or MDR *A baumannii* were recovered from the stainless steel and cotton carriers (>4–5 log_10_ reduction, depending on the starting inoculum). VHP was equally effective at all carrier locations. No difference in efficacy
Montazeri et al. 2017	USA	Inactivation of *human norovirus* after VHP exposure.	FCV. Outbreak *human NoV* GI.6 and GII.4.	AeroClave System 3110. 7.5% H_2_O_2_ liquid. No air handling unit during vapor process, at end of cycle turned on for 20 min. 7.1–15.9 mL/m^3^ was achieved, with 5 min dwell time following the manufacturer’s recommendation. Then the air handling unit was switched on for 20 min.	No blinding. No BI used. 7 locations in BSL-3 laboratory assessed with VHP. Inoculated coupons not exposed to VHP were outside the laboratory for the duration of the experiment.	Air-dried in a biosafety hood. And used immediately for the experiment. After the experiment, the samples were transferred to PBS tubes.	Data was determined to the control disc. This allows direct comparison to the test and control discs having the same log loss, making the comparison more accurate. But also leads to not knowing what the log loss of viral load is.	Stainless steel embossing tape	No trend was observed for *human NoV* GI.6 reduction as a function of H_2_O_2_-based disinfectant formulation concentration. However, increasing the concentration from 7.1 to 12.4 mL/m^3^ enhanced viral genomic copy number reduction for GII.4
Murdoch et al. 2016	United Kingdom	Assess the application of three different liquid concentrations for VHP.	MRSA and *Geobacillus stearothermophilus*	Bioquell BQ50 machine. 5, 10, and 35% H_2_O_2_. 640 g hydrogen peroxide over 40 min and 200 min dwell time.	No blinding labeled containers. BI used. Positive and negative controls. Every 10 min throughout the experiment a BI was exposed for 10 min.	All specimens were placed in labeled 30 mL containers.	No pathogens lost or contaminated samples were described or considered in the methodology. All data presented.	Stainless steel discs	35% hydrogen peroxide is ideal.
Otter et al. 2012	United Kingdom	Efficacy of VHP against methicillin-resistant Staphylococcus aureus on various surfaces.	MRSA	Bioquell Clarus R machine. No H_2_O_2_ liquid concentration. VHP mean concentration 134 ppm.	No blinding. No BI used. Control discs were not VHP exposed. The experiment ran in triplicate per period for each contaminant material.	Air-dried in the test room air, then VHP exposure and immediately enumerated.	No pathogens lost or contaminated samples were described or considered in the methodology. All data presented.	Stainless steel discs	Relative susceptibility to VHP was 10% BSA < TSB < 3% BSA = water. At a ppm achieved and >75 min exposure, no MRSA was recovered on the discs.
Petit et al. 2017	Brazil	Efficacy of VHP against *foot-and-mouth disease.*	Three serotypes of *Foot-and-mouth disease virus (FMDV)*	Bioquell Clarus R machine. 35% H_2_O_2_ liquid. 115 min. VHP injection time 75 min, 40 min dwell time.	No blinding. No validated BI manufactured by VHP producers. Positive controls of three serotypes. Three replicate cycles of 15 BI produced from FMDV for VHP exposure. Five samples for each viral serotype were produced. One plosive control per serotype for the duration of the experiment was stored in a refrigerator.	Dried in class 2 biological safety cabinet.	No pathogens lost or contaminated samples were described or considered in the methodology. All data presented.	Inside the cap of the polypropylene cryogenic tube.	Three *FMDV* serotypes showed full inactivation.
Pottage et al. 2012	United Kingdom	Comparison of log kill of BI vs MRSA after VHP exposure.	*G. stearothermophilus* and MRSA	A Steris VHP-1000ARD machine. 35% H_2_O_2_ liquid. 750 ppm maintained in chamber.	No blinding. Random removal of VHP exposed samples. 18 MRSA and 18 BI indicators placed in sterile Petri dishes and VHP exposed for pre-determined periods. Three unexposed stainless steel discs of each pathogen were used.	Inoculated stainless steel discs air-dried for 1 h.	No pathogens lost or contaminated samples were described or considered in the methodology. All data presented.	BI on stainless steel discs sealed in Tyvek packages.	BI greater log kill than MRSA for the same periods of exposure.
Pottage et al. 2019	United Kingdom	Efficacy of VHP on dried bacteria.	*Bacillus anthracis* (Ames) spores, *Brucella abortus*, Burkholderia pseudomallei, Escherichia coli, Mycobacterium *tuberculosis* and *Yersinia pestis*.	Bioquell Clarus C machine. 35% H_2_O_2_ liquid. 90 min cycle.	No blinding. Three controls tied in double plastic bags to determine the loss of log pathogen. 3 control samples were used as the start pathogen load. 12 produced BI for each VHP run to allow triplicate exposure. Three control BI from a VHP manufacturer used per VHP cycle.	Dried in a biological cabinet for 1 h.	No pathogens lost or contaminated samples were described or considered in the methodology. All data presented.	Stainless steel coupons in Petri dishes	This study demonstrates that VHP can inactivate a range of HG3 agents at high concentrations with associated organic matter, but *M*. *tuberculosis* showed increased resistance to the process.
Tuladhar et al. 2012	The Netherlands	Virucidal efficacy of VHP against respiratory and *enteric viruses* on various materials.	*Poliovirus, human norovirus* genogroup II.4 (GII.4), *murine norovirus* 1, *rotavirus, adenovirus*, and *influenza A* (H1N1) *virus.*	Boneco 7131 machine. 12% H_2_O_2_ liquid. 120–134 ppm at a flow rate of 2.3 L/h.	No validated BI manufactured by VHP producers. Triplicate samples per virus were performed twice. Control samples were not VHP exposed.	Dried in a biohazard cabinet.	No pathogens lost or contaminated samples were described or considered in the methodology. All data presented.	Stainless steel, framing panel, and gauze carriers.	VHP effective against pathogens assessed.
Wood et al. 2020	USA	Assess the decontamination efficacy of VHP on phages.	Bacteriophage viruses, MS2 and Phi6	Humidifier with 3 or 8% H_2_O_2_ liquid generated to 25 ppm. Bioquell Clarus C machine. 35% H_2_O_2_ liquid. 25 ppm and 400 ppm generated.	No validated BI manufactured by VHP producers. Inoculated samples, not VHP exposed, and inoculated samples VHP exposed. Two blank samples. Completed in triplicate.	Samples made and dried in a biosafety cabinet. After the experiment coupons were sealed and transferred to the biosafety cabinet.	No pathogens lost or contaminated samples were described or considered in the methodology. All data presented.	Stainless steel, glass, tile, N95 mask material, painted joint tape, wood.	Extrapolating from these results for both an enveloped and non-enveloped virus, we would expect LCHP would be a viable decontamination option for *EBOV* for relatively clean surfaces
